# How Human Subsistence Strategy Affected Fruit-Tree Utilization During the Late Neolithic and Bronze Age: Investigations in the Northeastern Tibetan Plateau

**DOI:** 10.3389/fpls.2022.941735

**Published:** 2022-07-01

**Authors:** Fengwen Liu, Hucai Zhang, Hu Li, Xiaonan Zhang, Qi Liu, Yang Zhang, Haoyu Li, Minmin Ma

**Affiliations:** ^1^School of Ecology and Environment Science, Institute for Ecological Research and Pollution Control of Plateau Lakes, Yunnan University, Kunming, China; ^2^School of History and Culture, Henan Normal University, Xinxiang, China; ^3^Ministry of Education Key Laboratory of Western China’s Environmental System, College of Earth and Environmental Sciences, Lanzhou University, Lanzhou, China

**Keywords:** charcoal analysis, fruit-tree utilization, subsistence strategy, northeastern Tibetan Plateau, Late Neolithic and Bronze Age

## Abstract

The history of fruit-tree utilization by prehistoric people has become an important issue that has attracted increasing attention in recent years. However, the question of how people used fruit trees has not yet been answered; in particular, the impacts of different subsistence strategies on human behavior regarding fruit-tree utilization (wild gathering or conscious cultivation) have not yet been considered. Here, we present the results of charcoal identification of fruit trees from 16 dated archeological sites in the northeastern Tibetan Plateau (NETP) spanning the period c. 5,200–2,600 BP. We combine this with reported multidisciplinary evidence to explore the history of fruit-tree utilization as well as its relation to the subsistence strategy in the NETP during the late Neolithic and Bronze Age. Our results demonstrate that Rosaceae [*Prunus* L., *Prunus Padus* L., *Maloideae* L., and *Malus baccata* (L.) *Borkh*], Elaeagnaceae (*Hippophae* L. and *Elaeagnus angustifolia* L.), and Rhamnaceae (only *Ziziphus* Mill.) were used by people in the NETP, and there was a downward trend in the use of fruit trees during the late Neolithic and Bronze Age. This is in notable contrast to the situation in the Chinese Loess Plateau in the parallel period. The cold-dry climate during the Bronze Age seemed to be one of the reasons. The fruit trees used by people in the NETP were likely gathered from the wild rather than consciously cultivated, and the subsistence strategy of agropastoralism may have played a significant role during the processes.

## Introduction

There is a Chinese proverb that states that “food is the first thing for people,” and in recent decades, archaeobotanists have focused on prehistoric crop and animal domestication as well as its impacts on human society, leading to some important results (Koca et al., 2006; Pearsall, 2008; Purugganan and Fuller, 2009; Tang et al., 2010; Zizumbo-Villarreal and Colunga-GarcíaMarín, 2010; Watling et al., 2018; Cubas et al., 2019; Liu and Reid, 2020). Diversified food selection since the Holocene played an important role in prehistoric humans’ adaptation to different environments, promoting the formation of diverse subsistence strategies around the world, such as hunting–gathering, agriculture, and agropastoralism (Matuzeviciute et al., 2020; Wang et al., 2020; Zhuang, 2020; Wang et al., 2021; Irish and Usai, 2021). Fruits have also been considered to be important edible resources throughout the history of human evolution (Eaton et al., 1997; Cordain, 2002; Zheng et al., 2014; Fuller and Stevens, 2019). However, it still remains unclear what changes have taken place in fruit trees utilization by people (wild gathering or conscious cultivation) since the prehistoric period. The question of whether different human subsistence strategies have affected fruit trees utilization appears to have been ignored, and it seems that it should be answered. Therefore, it is of great significance to consider study regions exhibiting the distribution of all kinds of fruit trees along with notable changes in human subsistence strategies. This will allow archaeological research on fruits to yield a nuanced understanding of the history of prehistoric human fruit domestication and/or cultivation.

The northeastern Tibetan Plateau (NETP) serves as an ideal region for exploring the changing processes of fruit trees utilization and its relationship to human subsistence. This is because: (1) an obvious transition in subsistence strategy has taken place in the NETP since the prehistoric period, moving from hunting–gathering (until 5,500 BP) to foxtail/broomcorn millet cultivation (5,500–3,600 BP) and later agropastoralism (wheat/barley cultivation and sheep herding) (3,600–2,300 BP) (Madsen et al., 2006; Rhode et al., 2007; Tang, 2011; Chen et al., 2015a; Zhang and Dong, 2017) (2) the history of the NETP as one of the important fruit-tree cultivation areas in China can be traced back to at least the Ming Dynasty (1,368–1,644 AD) (Yang, 2005), and cultivated fruit trees are still widely distributed across the NETP (Yang, 2005). Nevertheless, given the region’s scarcity of archaeobotanical evidence from the prehistoric period, the history of fruit trees utilization by people in the NETP is still unclear.

Recently, the remains of fruit stones unearthed from archeological sites have played a significant role in exploring the history of human behaviors on fruit trees domestication and/or cultivation during the prehistoric period, including the archeological sites of Shangshan, Pella, and Kuwait (Zheng et al., 2014; Dighton et al., 2017; Gros-Balthazard et al., 2017). Using flotation, a large quantity of plant remains have been collected from archeological sites in the NETP (Chen et al., 2015a); however, the fruit remains that have been discovered are less widely distributed, and this could lead to a chronological gap in the history of human fruit domestication or cultivation in the NETP. It could be inferred that the difficulty in the preservation of fossil plant remains alongside the seeds of the fruits in archeological sites has greatly limited the understanding of human fruit consumption and fruit trees utilization in the prehistoric period (Lee et al., 2007; Li, 2016; Halvorsen and Hjelle, 2017; Peña-Chocarro et al., 2019). Additionally, there has been a debate regarding the size criteria for certain species of domesticated fruits (Runnels and Hansen, 1986; Liphschitz and Bonani, 2001; Liphschitz et al., 2013; Dighton et al., 2017). This problem can be solved by charcoal analysis from fruit-tree remains in archeological sites (Wang et al., 2014, 2016; Asouti and Kabukcu, 2021; Moskal-del Hoyo, 2021), because a large number of archeological sites around the world contain charcoal from fruit trees that was left behind in a combustion process resulting from human management of fruit trees (Wang et al., 2014, 2016; Asouti and Kabukcu, 2021; Shen and Li, 2021; Slotten and Lentz, 2021). Therefore, analysis of charcoal from fruit trees in archeological sites can help with understanding the history of prehistoric fruit trees utilization by humans in the NETP.

Our aim in this study was to investigate the human utilization of fruit trees and its relationship with the different subsistence strategies that were adopted during the prehistoric period. We are especially interested in the changes in fruit-tree utilization in the NETP during the Late Neolithic and Bronze Age (5,200–2,600 BP), as there were remarkable changes in culture and human subsistence during that period (Madsen et al., 2006; Rhode et al., 2007; Tang, 2011; Chen et al., 2015a; Zhang and Dong, 2017). Charcoal identification and analysis—in conjunction with archaebotanical and zooarchaeological evidence and published paleoclimate records—were used to directly reveal what changes in fruit-tree utilization have occurred and their relationship with human subsistence in the NETP during the Neolithic and Bronze Age periods.

## Regional Setting

### Climate and Woody Vegetation

**FIGURE 1:**
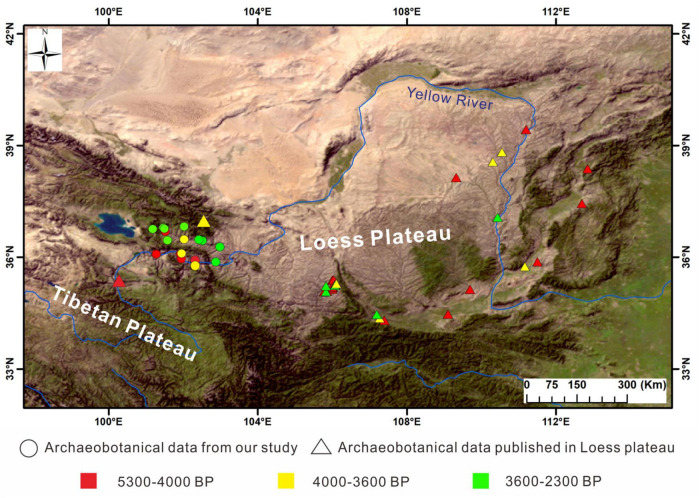
Distribution of archeological sites yielding charcoal analyses in the NETP and the Chinese Loess Plateau. Charcoal analysis sites presented in circles are located in the NETP (our new data), and those shown by triangles are in the Chinese Loess Plateau.

The terrain in the NETP is complex and has remarkable differences in altitude, and this leads to diversity in the climates of different areas (Figure 1). The annual average temperature in the Huangshui River basin (2,200 masl, above sea level) is 0.6–7.9^°^C, while that in the upper Yellow River valley is 2.1–8.5^°^C. Because of the higher altitude of the Huangshui River basin, the annual precipitation is about 50–100 mm higher than that in the upper Yellow River valley. These regional climatic differences lead to prominent spatial differences in woody vegetation in the NETP (China Forest Editorial Committee, 1997; Flora of China Editorial Committee, 2004). Influenced by the local hydrothermal conditions, needleleaf trees (including *Picea* L., *Pinus* L., *Abies*, *Larix*, and *Sabina przewalskii* Kom.) are usually distributed in the highlands at altitudes above 2,500 m because of the cold–humid climate, and the broadleaf trees (*Populus* L., *Salix* L., and *Betula*) and shrubs (*Tamarix* L., *Hippophae* L., etc.) grow in the valleys where the climate is warm and dry (China Forest Editorial Committee, 1997; Flora of China Editorial Committee, 2004). Additionally, the number of taxa of broadleaved trees shows a downward trend with elevation (China Forest Editorial Committee, 1997).

### Modern Fruit-Tree Cultivation

**FIGURE 2:**
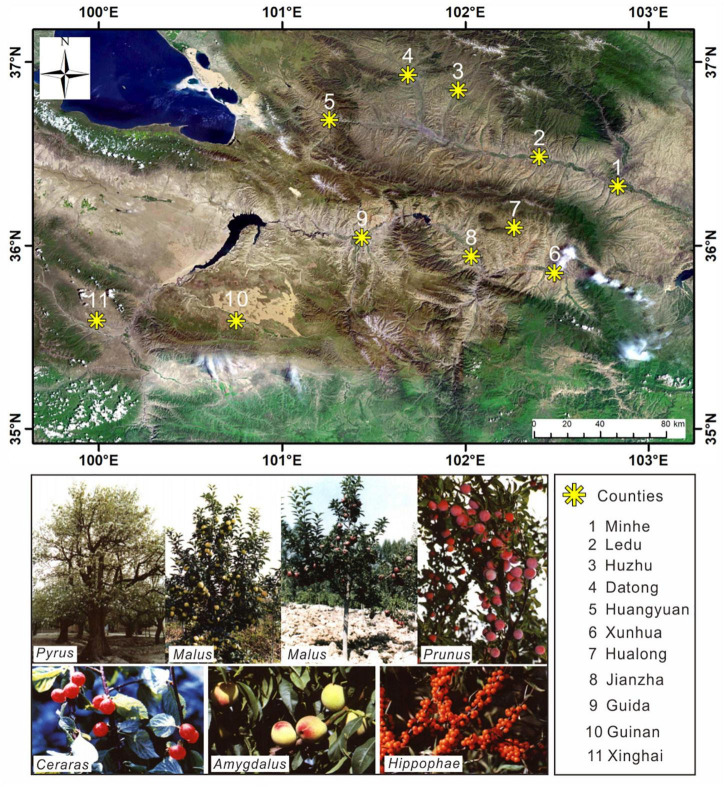
Distribution of modern fruit-tree types in the NETP. The yellow gear-shaped symbols represent the counties with fruit cultivation, including Minhe, Ledu, Guide, Huzhu, Xunhua, Hualong, Jianzha, Guinan, Datong, Xinghai, Huangyuan, and Huangzhong.

The Tibetan Plateau is currently one of the most important fruit-producing areas in China, and fruit-tree cultivation is mainly concentrated in its northeast. According to Yang (2005), the NETP produces large numbers of a range of fruits, including *Maloideae* L., *Pyrus* L., *Amygdalus* L., *Armeniaca* Mill., *Prunus pseudocerasus*, *Prunus* L., *Ziziphus* Mill., *Vitis vinifera*, and *Elaeagnus angustifolia* L. The altitudes of the areas that are suitable for fruit-tree planting falls in the range 1,600–3,100 masl (Figure 2). Additionally, there are many kinds of wild fruit resources distributed across the NETP, including *Hippophae* L., *Rosa multiflora* Thunb., *Elaeagnus angustifolia* L., *Sorbus pohuashanensis*, and *Cotoneaster* L. These wild fruit trees are distributed at high altitudes, usually above 2,500 masl, and they are considered to be a significant part of the forest ecosystem in the NETP.

### Cultural Evolution and Paleoeconomy

The NETP is one of the important areas for human colonization in the region. Early occupation in the NETP was by the Xiahe hominin population, which can be traced back to 190,000 BP (Chen et al., 2019; Zhang D. J. et al., 2020). Less human activity in the NETP took place in the period of the Last Glacial Maximum (25,000–18,000 BP). Evidence of foragers in the NETP can be seen again in the period 15,000–6,000 BP with their expansion to areas above 4,000 masl (Madsen et al., 2006; Rhode et al., 2007; Yi et al., 2011; Hou et al., 2016), and seasonal hunting–gathering was their main subsistence strategy (Tang, 2011; Wang et al., 2020).

Changes in the archeological culture in the NETP occurred in the Middle Holocene (about 5,500 BP), from Paleolithic to Neolithic Age (Majiayao culture, 5,300–4,000 BP) and Chalcolithic Age later (Qijia culture, 4,300–3,600 BP) (Xie, 2002). Humans mainly settled the areas below 2,500 masl in the NETP, and an agricultural economy was established in this period (Chen et al., 2015a). Around 4,000 BP, bronze wares were sporadically used by people in the NETP (Xie, 2002). During the period 3,600–2,300 BP, influenced by transcontinental cultural exchanges, the rapid development of agropastoralism facilitated human settlement toward to the areas above 2,500 masl in the NETP (Xie, 2002; Chen et al., 2015a). There were significant spatial differences in human subsistence strategies in the NETP during this period. In the areas below 2,500 masl, the people of Xindian culture (3,600–2,300 BP) were mainly engaged in crop cultivation, while in areas above 2,500 masl, those of the Kayue culture (3,600–2,300 BP) and Nuomuhong culture (3,400–2,300 BP) relied more on the use of pasture for activities such as sheep grazing (Xie, 2002; Zhang and Dong, 2017). The cultural attributes and human subsistence strategies of the sites investigated in this study are shown in Table 1.

**TABLE 1:** Radiocarbon dates of the investigated sites in the NETP during the late Neolithic and Bronze Age.

	Site	Materials	Lab. no.	Radiocarbon age (yr BP)	Calibrated age (cal yr BP) 2 sigma	Culture	Human subsistence	References
5,300–4,000 BP	Luowalinchang	Broomcorn millet	BA 120197	4,470 ± 25	5,141 ± 164	Majiayao	Agriculture	Chen et al., 2015a
	Gayixiangjing	Foxtail millet	Beta-297655	4,410 ± 40	5,068 ± 206	Majiayao	Agriculture	Chen et al., 2015a
	Benbakou	Foxtail millet	BA 110909	4,185 ± 25	4,731 ± 105	Majiayao	Agriculture	Chen et al., 2015a
	Shangduoba	Broomcorn millet	BA 120187	4,035 ± 30	4,600 ± 178	Majiayao	Agriculture	Chen et al., 2015a
	Shangsihesheng	Charcoal	LUG10-187	3,985 ± 51	4,519 ± 261	Majiayao	Agriculture	Jia, 2012
	Early Gongshijia	Charcoal	LUG11-64	3,802 ± 50	4,206 ± 201	Qijia	Agriculture	Jia, 2012
	Zongri	Foxtail/broomcorn millet	4,600–4,000			Zongri	Hunting-gathering	Ren, 2017
4,000–3,600 BP	Jinchankou	Barley	BA 110913	3,595 ± 20	3,906 ± 65	Qijia	Agriculture	Chen et al., 2015a
	Middle Gongshijia	Barley	Beta-303689	3,620 ± 30	3,955 ± 112	Qijia	Agriculture	Chen et al., 2015a
	Dongcun*		4,060–3,580			Qijia	Agriculture	Jia, 2012
	Late Gongshijia	Charcoal	LUG11-62	3,413 ± 49	3,672 ± 159	Qijia	Agriculture	Jia, 2012
3,600–2,600 BP	Wenjia	Barley	BA 110888	2,890 ± 30	3,041 ± 115	Xiandian	Agro-pastoralism	Chen et al., 2015a
	Wayaotai	Broomcorn millet	BA 120199	3,410 ± 30	3,694 ± 121	Xiandian	Agro-pastoralism	Chen et al., 2015a
	Shuangerdongping	Barley	BA 110903	2,770 ± 25	2,867 ± 78	Xiandian	Agro-pastoralism	Chen et al., 2015a
	Xigang	Charcoal	LUG11-138	2,583 ± 66	2,612 ± 235	Xiandian	Agro-pastoralism	Jia, 2012
	Qiakadingdong*		3,140–3,060			Kayue	Agro-pastoralism	Jia, 2012
	Dingke*		3,140–3,060			Kayue	Agro-pastoralism	Jia, 2012
	Kasuo*		3,140–3,060			Kayue	Agro-pastoralism	Jia, 2012
	Shangyagen*		3,140–3,060			Kayue	Agro-pastoralism	Jia, 2012
	Miaogou*		3,140–3060			Kayue	Agro-pastoralism	Jia, 2012

The chronology of the Site* are confirmed by the radiocarbon dates of other archeological sites in corresponding culture. Ages obtained were calibrated using Calib (v.6.0.1) (Stuiver and Reimer, 1993) and the IntCal09 calibration curve (Reimer et al., 2009). They are presented as cal yr BP with two sigma (at 95.4% confidence).

## Materials and Methods

### Sites Investigated and Materials Analyzed

**FIGURE 3:**
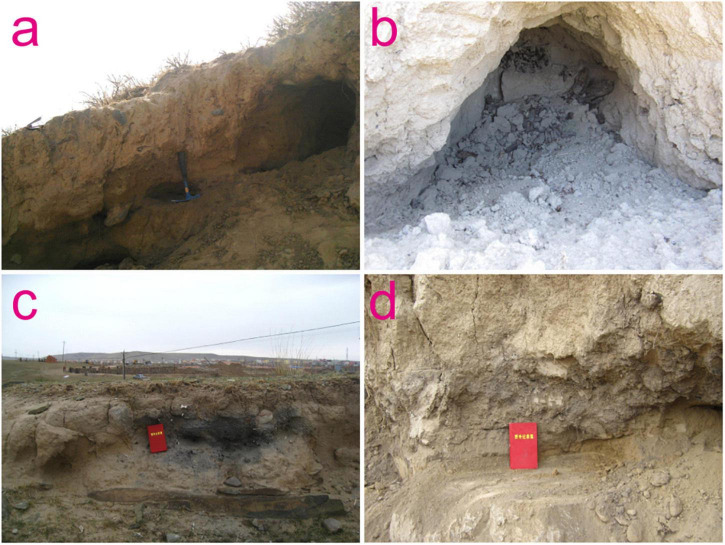
Photographs of charcoal remains collected from some of the investigated sites in the NETP. **(a)** Luowalinchang; **(b)** Gayixiangjing; **(c)** Gongshijia; **(d)** Wenjia.

A total of 16 archeological sites in the NETP were investigated, and these were distributed along the Huangshui River and the upper reaches of the Yellow River. People in the investigated sites of Majiayao culture and Qijia culture were engaged in an agricultural economy, and those of Xindian culture and Kayue culture were engaged in agropastoralism (Table 1). The archeological contexts of the soil samples were all exposed sections inside the archeological sites, with charcoal and ceramic remains embedded, and these were from exposed fills of cultural layers and ash pits (Figure 3). A total of 155 soil samples were collected, with each of these processed by the flotation method. The soil was washed in a bucket over a #80-mesh sieve (aperture size of 0.2 mm) to gather any carbonized remains. The collected carbonized remains were dried in the shade and then sorted. Pieces of charcoal with a diameter ≥ 2 mm were chosen using sieves with apertures of 4, 2, 1, 0.7, and 0.35 mm. Their microscopic features were then examined, and taxonomic species were determined using a metallurgical microscope in the MOE Key Laboratory of Western China’s Environmental Systems at Lanzhou University. A high-resolution primary atlas of modern wood specimens was used to determine the taxa of charcoal remains, and the those of fruit trees were selected (Cheng et al., 1992). The calculation of relative percentages was carried out to explore the changes in fruit-tree assemblages in the NETP during the Late Neolithic and Bronze Age. To explore human utilization of fruit trees in the study area, we compared our results with those from the Chinese Loess Plateau, where conscious fruit-tree cultivation occurred in parallel periods.

### Radiocarbon Dates of the Archeological Sites

As shown in Table 1, the chronologies of the archeological sites investigated were conducted and reported by Chen et al. (2015a) (*n* = 10; foxtail millet, broomcorn millet, and barley as the dating samples), Ren (2017) (*n* = 10; foxtail millet and broomcorn millet, as the dating samples) and Jia (2012) (*n* = 4; charcoal as the dating samples; *n* = 6, relative chronology from the ceramic and radiocarbon dates of other cultural ruins). The dates of archeological sites discussed are relative to AD 1950 (referred to as “BP”).

## Results

We identified 3,981 charcoal remains from 16 archeological sites in the NETP (Table 2 and Figure 1). Collected samples were separated into three chronological periods based on the differences in human subsistence strategies in the NETP: Neolithic Age (5,500–4,000 BP), Chalcolithic Age (4,000–3,600 BP), and Bronze Age (3,600–2,300 BP). Seven samples were collected from Majiayao and Qijia culture with dates of 5,200–4,000 BP; 132 samples came from Zongri culture dating from 4,600 to 4,000 BP (Liu et al., 2021); four samples were from Qijia culture, dated between 4,000 and 3,600 BP. The archeological sites of the Bronze Age were investigated and found to contain Xindian culture and Kayue culture dating from 3,600 to 2,600 BP, of which nine samples were Xindian and five were Kayue.

**TABLE 2:** Absolute counts and relative percentages of the fruit trees by charcoal analysis in the investigated sites in the NETP during the late Neolithic and Bronze Age.

	Site	Sampling sites	Soil samples count *n*/soil *L*	*Prunus* L. count *n*/rel.%	*Prunus padus* L. count *n*/rel.%	*Maloideae* L. count *n*/rel.%	*Malus baccata* (L.) *Borkh* count *n*/rel.%	*Subg. Persica* L count *n*/rel.%	*Pyrus* L. count *n*/rel.%	*Elaeagnus angustifolia* L. count *n*/rel.%	*Ziziphus* Mill count *n*/rel.%	*Hippophae* L. count *n*/rel.%	Identified wood fragments (total number)	References
5,300–	Luowalinchang	Cultural layer	1/10	–	–	8/2.25%	–	–	–	–	–	84/23.6%	356	This study
4,000	Gayixiangjing	Ash pits	1/10	–	–	–	–	–	–	–	–	51/50%	102	This study
BP	Benbakou	Cultural layer	1/10	–	–	18/17.65%	–	–	–	–	–	–	102	This study
	Shangduoba	Cultural layer	1/10	6/6%	2/2%	8/8%	–	–	–	–	–	22/22%	100	This study
	Shangsihesheng	Cultural layer	1/10	1/1%	1/1%	3/3%	–	–	–	–	–	7/7%	100	This study
	Early Gongshijia	Cultural layer	2/20	4/1.49%	2/0.75%	3/1.12%	–	–	–	–	–	23/8.58%	268	This study
	Zongri	Cultural layer	132/235	–	–	–	–	–	–	–	–	462/22.61%	2,043	Liu et al., 2021
4,000–	Jinchankou	Cultural layer/Ash pit/House/Kiln	120/	–	–	–	–	14/1.36%	1/0.1%	2/0.19%	–	171/16.63%	1,028	Wang et al., 2016
3,600	Gongshijia	Cultural layer	1/10	12/1.37%	12/1.37%	17/1.94%	14/1.59%	–	–	–	–	103/11.73%	878	This study
BP	Dongcun	Cultural layer	1/10	–	–	–	–	–	–	–	–	8/5.63%	142	This study
3,600–2,600 BP	Wenjia	Cultural layer	3/30	4/1.65%	–	2/0.82%	–	–	–	–	–	30/12.35%	243	This study
	Wayaotai	Ash pits	1/10	2/1.71%	3/2.56%	12/10.26%	–	–	–	–	–	4/3.42%	117	This study
	Shuangerdongping	Cultural layer	4/40	–	–	19/3.25%	–	–	–	3/0.51%	–	6/1.03%	585	This study
	Xigang	Ash pits	1/10	–	3/2.78%	8/7.41%	–	–	–	–	–	–	108	This study
	Qiakadingdong	Cultural layer	1/10	–	–	1/1%	–	–	–	–	–	2/2%	100	This study
	Dingke	Cultural layer	1/10	1/0.5%	–	–	–	–	–	–	–	1/0.5%	200	This study
	Kasuo	Cultural layer	1/10	–	6/2.93%	8/3.9%	–	–	–	1/0.49%	–	58/28.29%	205	This study
	Shangyagen	Ash pits	1/10	4/2%	–	22/11%	–	–	–	–	1/0.5%	–	200	This study
	Miaogou	Cultural layer	1/10	–	1/0.46%	–	–	–	–	–	–	–	218	This study

According to the relationship between human wood use and the accounts of charcoal pieces identified in different archeological sites, there are clear differences in the standards required to obtain accurate reflections for different regions (Keepax, 1988; Scheel-Ybert, 2002; Li et al., 2012). In temperate regions, a minimum of 100 charcoal fragments per sample should be identified to provide a good representation of most types of charcoal (Li et al., 2012; Liu et al., 2021). In our study, the number of charcoal fragments identified in each sample reached or exceeded 100, aside from a single sample from the Zhongtan site. Therefore, the result from this site was not included in subsequent analysis.

**FIGURE 4:**
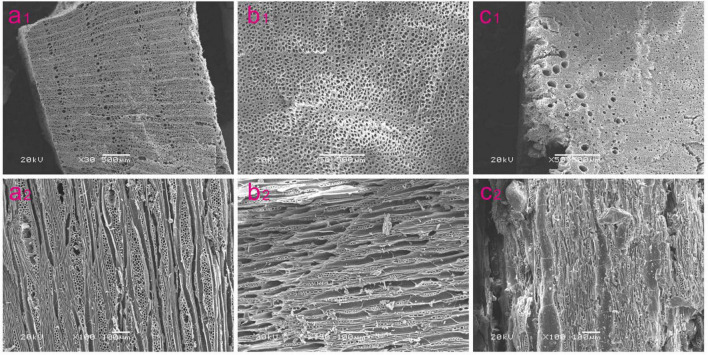
Charcoal remains of fruit trees identified from the investigated sites in the NETP. **(a_1_,a_2_)** *Prunus* L.; **(b_1_,b_2_)** *Maloideae* L*.;* **(c_1_,c_2_)**, *Hippophae* L., quoted from Liu et al. (2022).

For comparative analysis, the results of the charcoal identification of fruit trees can be conveniently divided into three different periods, as detailed above. During the Late Neolithic (5,500–4,000 BP), a total of four taxa of fruit trees were identified—*Prunus* L., *Prunus padus* L., *Maloideae* L., and *Hippophae* L.—of which the relative percentage of *Hippophae* L. was the highest in the fruit-tree assemblage and that of *Maloideae* L. was the second highest. Only *Hippophae* L. was found in the site of Zongri culture (Liu et al., 2021). Eight taxa of fruit trees were found in the investigated sites during the Chalcolithic (4,000–3,600 BP). The fruit trees of *Malus baccata* (L.) *Borkh*., subg. *Persica* L., *Pyrus*, and *Elaeagnus angustifolia* L. appeared most commonly in the investigated sites and Jinchankou site (Wang et al., 2016). There was a similar situation in the Late Neolithic, and the relative percentage of *Hippophae* L. was still the largest. During the Bronze Age (3,600–2,300 BP), six taxa of fruit trees were identified: *Prunus* L., *Prunus padus* L., *Maloideae* L., *Ziziphus* Mill., *Hippophae* L., and *Elaeagnus angustifolia* L. The proportion of *Hippophae* L. showed an obvious decrease in the investigated sites, being replaced by *Maloideae* L. as the most abundant in the fruit-tree assemblage of the Xindian culture. For the complete charcoal-identification data of fruit trees from each period (see Table 2 and Figure 4).

## Discussion

### History of Fruit Trees in Human Use in the Northeastern Tibetan Plateau and Its Differences From the Loess Plateau During the Late Neolithic and Bronze Age

The utilization of fruit trees has attracted much attention from an archaeobotanical perspective (Terral, 1996; Li et al., 2012; Zheng et al., 2014; Dotte-Sarout, 2017; Moskal-del Hoyo, 2021; Shen and Li, 2021). Our results demonstrate that the fruit trees that were used by people in the NETP during the Late Neolithic and Bronze Age (5,200–2,600 BP) included Rosaceae (*Prunus* L., *Padus* Mill., *Maloideae* L., and *Malus* Mill.), Elaeagnaceae (*Hippophae* L. and *Elaeagnus angustifolia* L.), and Rhamnaceae (only *Ziziphus* Mill.). Charcoal evidence from the Jinchankou site suggested that humans also gathered the trees of subg. *Persica* L. and *Pyrus* L. during the Late Neolithic (Wang et al., 2016). Among these tree species, *Hippophae* L., and *Maloideae* L. were the main fruit trees used by people in the NETP both in the Late Neolithic and the Bronze Age, while the relative percentage of *Maloideae* L. showed a significant uptrend across the duration of the Bronze Age and exceed that of *Hippophae* L. in the group of Xindian culture. Other fruit trees, including *Prunus* L., *Padus* Mill., *Malus* Mill., *Elaeagnus angustifolia* L., and *Ziziphus* Mill., were also in use in the NETP during the Late Neolithic and Bronze Age, but their lower relative percentages indicate that they were not the dominant species (Table 2).

Some archaeobotanical evidence has shown that the domestication of fruits can be traced back to the early and middle Holocene, such as the domesticated peach in China (Zheng et al., 2014), the domesticated grape in Greece (Pagnoux et al., 2021), and the olive in the Mediterranean region (Terral, 1996). Chinese historical documents such as *Shijing* (the *Classic of Poetry*) also indicate that fruit trees, taking apricot (*Prunus* L.) and jujube (*Ziziphus* Mill.) as examples, were brought into cultivation in China from the fourth millennium BP. Therefore, the question arises as to whether the fruits trees presented in the NETP were gathered by people from the wild or were consciously cultivated. To consider this question, we should first examine whether the fruit trees discovered in the investigated sites were from the local area. If the fruit trees in the investigated sites were collected from long distances, either by trade or cultural exchange, this could lead to ambiguous conclusions; for example, since the prehistoric, domesticated fruits for cultivation have been introduced to western Europe from long distances (Asouti and Kabukcu, 2014; Pérez-Jordà et al., 2021). According to Yang (2005), many fruit trees are widely cultivated in the NETP, including *Maloideae* L., *Pyrus* L., *Amygdalus* L., *Armeniaca* Mill., *Prunus pseudocerasus*, *Prunus* L., *Ziziphus* Mill., *Vitis vinifera*, and *Elaeagnus angustifolia* L., and the altitudes of this cultivation fall into the range 1,600–3,100 masl (Figure 2). This indicates that the fruit trees discovered in our study could fit with the environment and be widely distributed in the NETP. Shackleton and Prins (1992) proposed that human wood-gathering in the prehistoric period followed the principle of least effort, and from the model it can be considered that there will be consistency between the situation of the fruit trees used by people and the distribution of modern fruit trees in the NETP. Therefore, the fruit trees discovered in the investigated sites in the NETP were likely obtained from people’s immediate habitats.

**FIGURE 5:**
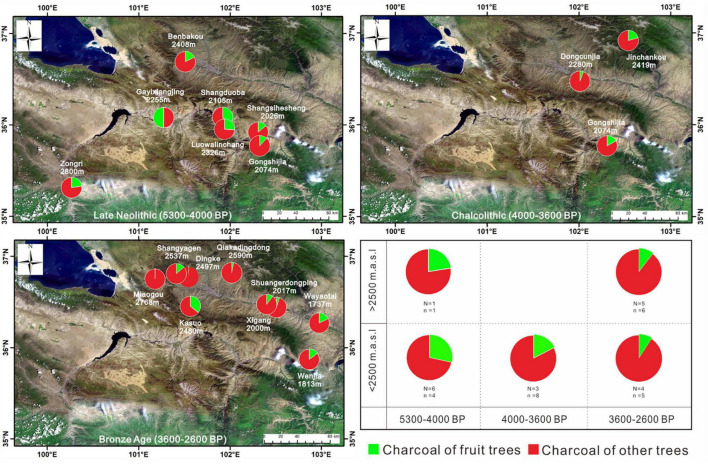
Spatiotemporal comparisons of woody assemblages (including the proportions of fruit trees and other woody plants) from the investigated sites in the NETP dated to the periods 5,300–4,000 BP, 4,000–3,600 BP, and 3,600–2,600 BP (*n* is the number of fruit-tree taxa identified; *N* is the number of investigated sites).

The utilization of fruit trees, from a certain species’ domestication to intensive cultivation, is likely to have taken thousands of years (Miller and Gross, 2011). In China, the domestication of fruits such as peach can be traced back to 8,000 BP, and conscious selection of preferred types in these fruits occurred in the Yangtze River valley during that period (Zheng et al., 2014). However, the intensive cultivation of fruit trees in China probably occurred during the Bronze Age (Shen and Li, 2021). Humans in the Chinese Loess Plateau have consciously cultivated fruit trees and collected their fruits as luxury food for feasting since the fourth millennium BP (Li, 2016, p. 32; Shen and Li, 2021), and this has been confirmed by Chinese historical documents such as *Shijing*, *Shanhaijing* (the *Classic of Mountains and Seas*), and *Xiaxiaozheng* (a record of traditional agriculture in China both during the prehistoric and historical periods). Archaeobotanical evidence from the Chinese Loess Plateau shows that the relative percentage of fruit-tree charcoal increased significantly during the Bronze Age in comparison with the Late Neolithic (Li, 2016; Shen and Li, 2021). A possible reason for this might be the increased frequency of artificial pruning of the branches and forks of fruit trees as well as fruit collection, which led to an increase in both the relative percentage and ubiquity of fruit-tree charcoal in archeological sites (Salavert and Dufraisse, 2014). However, a reverse trend occurred in the NETP whereby the relative percentage of fruit-tree charcoal clearly decreased from the Late Neolithic to the Bronze Age (Figures 4, 5). Additionally, there is less evidence suggesting the presence of fruit seeds or stones in archeological sites in the NETP during this period. This demonstrates that the use of fruit trees by people in the NETP gradually decreased from the Late Neolithic to the Bronze Age.

### Factors Affecting Human Utilization of Fruit Trees in the Northeastern Tibetan Plateau During the Late Neolithic and Bronze Age

In general, the growth of trees, together with forest changes, is closely related to the climate. The records of forest changes due to climate alteration throughout the Holocene have been widely reported (Li et al., 2017; Rita et al., 2018; Schiferl et al., 2018; Han et al., 2019; Zhang D. L. et al., 2020). Despite their lower prevalence, which was always at a low level in terms of proportion of the forest, the dynamics of fruit trees was still affected by the local climate. According to modern observation records, a cooler or drier climate is not conductive to the growth of fruit trees, and this will lead to a decrease in their distribution as well as in fruit production in northwest China (Yao et al., 2005; Peng et al., 2018). The spatial differences in climate in the NETP are obvious, and the average annual temperature decreases with altitude. For instance, the average annual temperature in the Huangshui River basin is a little lower than that in the upper reaches of the Yellow River valley, and the relatively higher altitude of the former plays an important role in this. However, the human use of fruit trees was not found to present obvious spatial differences.

**FIGURE 6:**
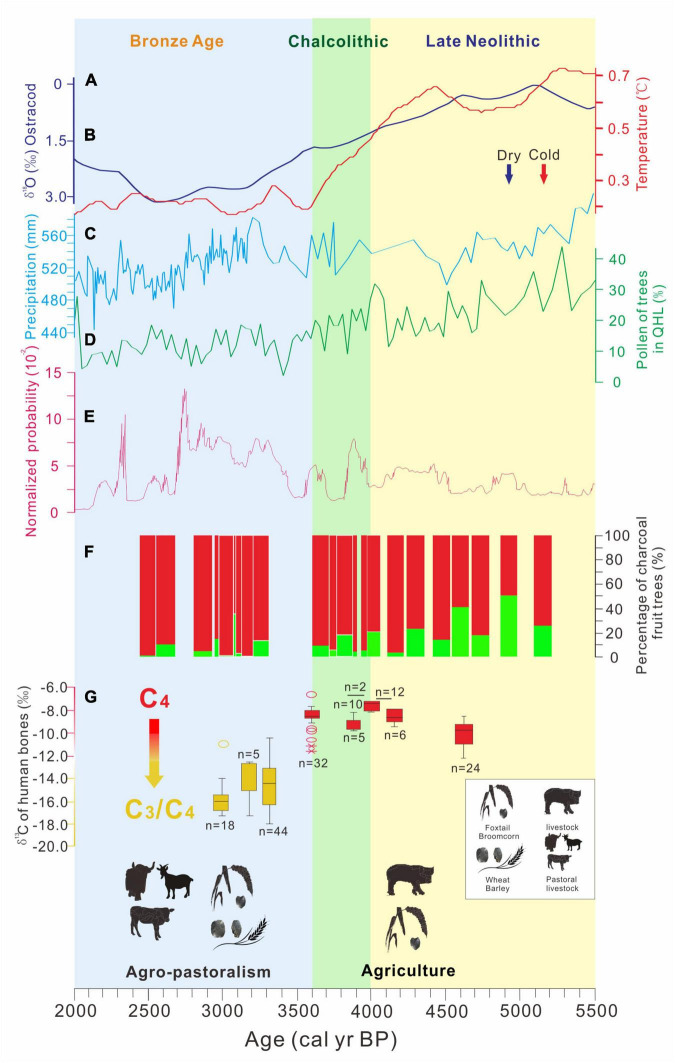
Relationships between climate change, human subsistence, and proportions of fruit trees. **(A)** Quantitative reconstruction of Holocene temperature changes in the Northern Hemisphere (Marcott et al., 2013); **(B)** results of stable oxygen isotope measurements performed on ostracod values in sediment cores of Lake Qinghai (Liu X. Q. et al., 2007); **(C)** quantitative reconstruction of Holocene precipitation changes in northern China (Chen et al., 2015b); **(D)** pollen record of trees in sediment cores of Lake Qinghai (Liu et al., 2002); **(E)** normalized probability from radiocarbon dates of archeological sites in the NETP (5,500–2,000 BP); **(F)** proportions of fruit trees in the investigated sites in the NETP (green rectangles); **(G)** stable carbon isotope ratios (δ ^13^C) of human bones in the NETP and the adjacent Chinese Western Loess Plateau (Chen et al., 2015a).

**FIGURE 7:**
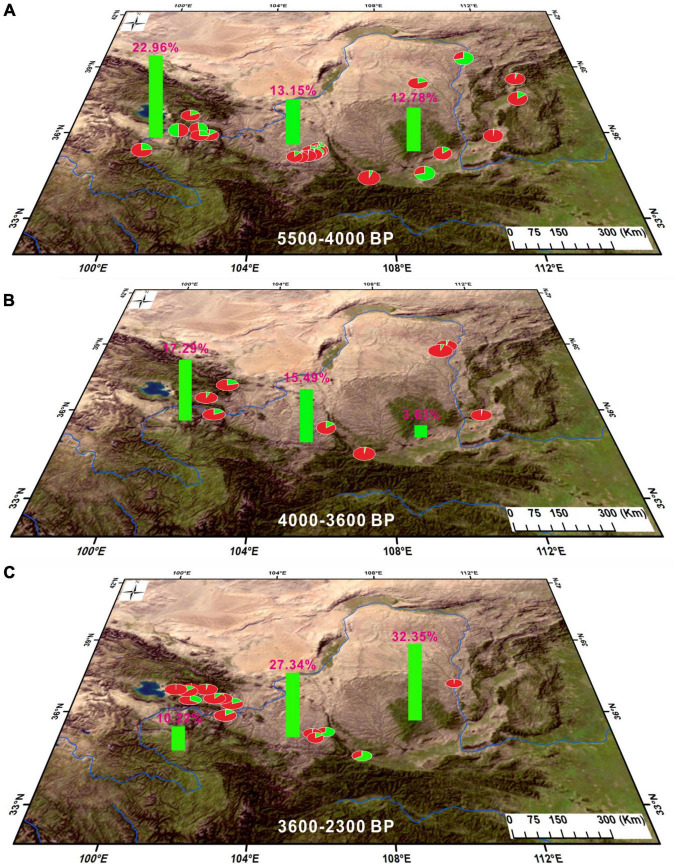
Spatiotemporal comparisons of woody assemblages (including the proportions of fruit trees and other woody plants) in the NETP and Chinese Loess Plateau dated to the periods 5,500–4,000 BP, 4,000–3,600 BP, and 3,600–2,300 BP. Green indicates the proportion of fruit trees and red indicates the proportion of other trees.

The proportion of fruit trees was similar in the Xindian culture (below 2,500 masl) and the Kayue culture (near and above 2,500 masl), with relative proportions of 9.12 and 10.46%, respectively (Table 2 and Figure 5). Thus, the human use of fruit trees was not affected by the spatial differences in climate in the NETP. In terms of temporal patterns, there was a period of cooling and drying climate and a corresponding decline in tree pollen from lakes both in the NETP and the Chinese Loess Plateau during the Late Neolithic and Bronze Age (Figure 6; Liu et al., 2002, Liu X. Q. et al., 2007; Sun, 2011; Marcott et al., 2013; Chen et al., 2015b). Nevertheless, the percentages of fruit trees in the Chinese Loess Plateau increased in the parallel period (Figure 7), which crucially demonstrates that humans were consciously cultivating fruit trees, investing time in management to withstand the impact of unfavorable climate conditions (Li, 2016; Shen and Li, 2021). In contrast, the cooling and drying climate since the Late Neolithic was probably responsible for the decline in the human use of fruit trees in the NETP. This seems to indicate that people did not consciously cultivate fruit trees with aborative management strategies in the NETP during this period.

Archeological evidence indicates obvious temporal variation in human behavior regarding fruit-tree management during the prehistoric period. In the Paleolithic, fruits served as significant foods for human survival (Liu L. et al., 2007; Jones and Liu, 2009). The lower prevalence of dental caries in people during the Paleolithic could be crucial evidence for this (Koca et al., 2006). Due to human subsistence by hunting–gathering, which is a strategy that involves high mobility, edible fruits were usually gathered from the wild, and no fruit-tree cultivation occurred during this period. At the initial stage of cereal domestication around 10,000 BP, people still regarded wild fruits as an important food supply. This is because the subsistence strategy of hunting–gathering initially continued to be dominant in the social economy (Fuller and Qin, 2010; Zhang et al., 2018). Archaeobotanical evidence has shown that the proportion of carbonized fruit seeds/stones in some archeological sites was far greater than that of cereals, for example in the Bancun, Jiahu, and Zhuzhai sites (Zhao and Zhang, 2009; Bestel et al., 2017; Zhang et al., 2018).

With the intensification of agriculture in the middle and late Holocene, an agriculture economy with crop cultivation and domesticated animal breeding was established in northern China (Zhao, 2007, 2014, 2017). The abundance of foxtail/broomcorn millet consumed by people likely led to a downward trend in wild fruit resources in the food supply and the use of fruit trees, and this is confirmed by evidence that the percentages and ubiquities of fruit-tree charcoal in the Chinese Loess Plateau during the Late Neolithic were much lower in comparison with those during the Early Neolithic (Figure 7; Li, 2016; Shen and Li, 2021). However, remarkable differences indicate that the use of fruit trees was high in the NETP during 5,500–4,000 BP. The relative percentage of fruit-tree charcoal in each of the investigated sites was no less than 12%, and it was significantly higher than that in the Chinese Loess Plateau (Table 2 and Figure 7). This could be attributed to the adoption of a special subsistence strategy in the NETP during the Late Neolithic. Foxtail/broomcorn millet cultivation was adopted by people in the NETP around 5,500 BP, and the exploitation of animals for meat was mainly from wild animals such as Cervidae, Caprinae, and Antilopinae whose relative percentage were no less than 60% (Ren, 2017), which indicates that hunting–gathering was still important in human subsistence in the NETP during the Late Neolithic. Thus, the fruit trees presenting in the NETP during this period were likely gathered from the wild in the process of wild-animal hunting.

Archaeobotanical evidence from different regions of the world mainly points to the fact that the conscious human cultivation of fruit trees, such as jujube, apricot, and grape, began around the fourth millennium BP or even earlier (Pagnoux et al., 2021; Shen and Li, 2021). In regions of fruit-tree cultivation, human society has undergone obvious changes since the Bronze Age, including the emergence of urbanization, craft specialization, and even commodity specialization in wine and dried fruits (Pérez-Jordà et al., 2021; Shen and Li, 2021). These changes can be attributed to the establishment of a settled agricultural economy. Archeological evidence from the Chinese Loess Plateau suggests a prosperous society with abundant harvests of all food crops and thriving breeding of domesticated animals during the Bronze Age (Zhao, 2011). This could have provided favorable conditions for handicraft and trade and promoted the cultivation of fruit trees. This is also evidenced by the increasing trend of the percentages and ubiquities of fruit remains as well as charcoal presented in archeological sites located in the Chinese Loess Plateau during the Bronze Age (Figure 7; Li, 2016; Shen et al., 2021).

In contrast, an agropastoral subsistence strategy gradually developed in the NETP during the Bronze Age (Chen et al., 2015a; Zhang and Dong, 2017). Influenced by cultural exchange across Eurasia, wheat/barley cultivation and sheep grazing were adopted by people in the NETP (Figure 6; Chen et al., 2015a; Zhang and Dong, 2017). Zooarchaeological evidence indicates that domesticated animals suitable for grazing—such as sheep, cattle, and horses—were much more abundant than pigs in archeological sites and tombs of the Xindian, Kayue, and Nuomuhong cultures (Dong et al., 2016; Zhang and Dong, 2017). This indicates that grazing behavior was likely dominant in human subsistence in the NETP during the Bronze Age. This subsistence strategy has relatively high mobility, which is not conducive to fruit-tree cultivation and fruit production. In the Keep River region of Australia, it has been demonstrated that the decline of fruit-tree cultivation and fruit consumption can be attributed to the rise of pastoralism (Atchison et al., 2005). Therefore, the sharp decline in fruit-tree utilization in the NETP could be responsible for their subsistence strategy during the Bronze Age, and the fruit-tree samples in the investigated sites were thus not likely to have been gathered from cultivated trees.

## Conclusion

Using analysis of charcoal from archeological sites, we examined the history of fruit-tree management by people in the NETP during the Late Neolithic and Bronze Age (5,500–2,300 BP). The fruit trees used by people were almost all from the local area, including Rosaceae [*Prunus* L., *Prunus Padus* L., *Maloideae* L., and *Malus baccata* (L.) *Borkh*], Elaeagnaceae (*Hippophae* L. and *Elaeagnus angustifolia* L.), and Rhamnaceae (only *Ziziphus* Mill.). There was a downward trend in the human use of fruit trees during the Late Neolithic and Bronze Age. The cold-dry climate during the Bronze Age seemed to be one of the reasons. However, this is in notable contrast to the trend in the Chinese Loess Plateau in the parallel period, during which time conscious cultivation of fruit trees took place. The results indicate that the fruit trees used by people in the NETP were likely gathered from the wild rather than consciously cultivated. The consequent adoption of agropastoralism during the Bronze Age may have played a significant role in this. This study provides new evidence for the in-depth understanding of prehistoric human use of plant resources in the NETP, and it gives a significant scientific basis for the changing process of past human management of fruit trees from the perspective of differences in human subsistence strategies.

## Data Availability Statement

The raw data supporting the conclusions of this article will be made available by the authors, without undue reservation.

## Author Contributions

HZ and MM conceived the study. FL undertook the identification of charcoal remains and wrote the manuscript. HL, XZ, QL, YZ, and HYL discussed the data. All authors contributed to the article and approved the submitted version.

## Conflict of Interest

The authors declare that the research was conducted in the absence of any commercial or financial relationships that could be construed as a potential conflict of interest.

## Publisher’s Note

All claims expressed in this article are solely those of the authors and do not necessarily represent those of their affiliated organizations, or those of the publisher, the editors and the reviewers. Any product that may be evaluated in this article, or claim that may be made by its manufacturer, is not guaranteed or endorsed by the publisher.
